# Comparison of the Effect of and Compliance With Cyclosporine 0.1% After Various Pretreatments in Dry Eye Disease

**DOI:** 10.1155/joph/6744482

**Published:** 2025-01-07

**Authors:** Donghyun Jee, Su Yeon Han, Hyun Seung Kim, Eun Chul Kim

**Affiliations:** ^1^Department of Ophthalmology, St. Vincent's Hospital, College of Medicine, Catholic University of Korea, Suwon, Republic of Korea; ^2^Department of Ophthalmology, Bucheon St. Mary's Hospital, College of Medicine, Catholic University of Korea, Bucheon, Republic of Korea; ^3^Department of Ophthalmology, Seoul St. Mary's Hospital, College of Medicine, Catholic University of Korea, Seoul, Republic of Korea

**Keywords:** cyclosporine 0.05%, cyclosporine 0.1%, fluorometholone 0.1%, Sjögren's syndrome

## Abstract

**Purpose:** We sought to compare the effect of cyclosporine 0.1% after various pretreatments in patients with dry eye disease.

**Methods:** Two hundred seventy-four eyes of 137 patients diagnosed with dry eye disease were retrospectively enrolled. Thirty patients (Group 1, 60 eyes) were not pretreated, while 68 patients (Group 2, 136 eyes) were pretreated with fluorometholone 0.1%, and 39 patients (Group 3, 78 eyes) were pretreated with cyclosporine 0.05% before treatment with cyclosporine 0.1%. The Ocular Surface Disease Index Questionnaire (OSDI) score, Schirmer I test result, noninvasive tear film break-up time (NItBUT), corneal staining score, matrix metalloproteinase-9 (MMP-9) grade, meibography result, meibum quality and expressibility scores, and tear meniscus height were examined before treatment and at 1, 2, and 3 months after treatment.

**Results:** All dry eye signs and symptoms of all Groups at 1, 2, and 3 months were significantly improved compared to those before treatment with cyclosporine 0.1% (*p* < 0.05). Notably, the OSDI score, Schirmer I test result, NItBUT, corneal and conjunctival fluorescein score, and MMP-9 grade in Group 3 were significantly improved compared to those in Groups 1 and 2 at 1, 2, and 3 months after treatment with cyclosporine 0.1% (*p* < 0.05). The percentages of cases with treatment discontinuation in Groups 1, 2, and 3 were 20.0%, 7.4%, and 10.0%, respectively.

**Conclusion:** Pretreatment with cyclosporine 0.05% can augment the anti-inflammatory effect of cyclosporine 0.1%. Pretreatment with a steroid or a lower concentration of cyclosporine can increase compliance in patients using a cyclosporine 0.1% eye drop.

## 1. Introduction

The Dry Eye Workshop II has published a new definition of dry eye disease (DED), defining it as a multifactorial disease of the ocular surface characterized by loss of homeostasis of the tear film accompanied by ocular symptoms in which tear film instability and hyperosmolarity, ocular surface inflammation and damage, and neurosensory abnormalities play etiological roles [1]. Because inflammation is crucial in the pathogenesis of DED, many anti-inflammatory eye drops have been produced [2].

Topical cyclosporine is one of the useful pharmacologic treatments available for patients with DED. Following treatment with cyclosporine 0.05% in patients with DED, improvements in blurry vision, decreased frequency of artificial tear use, reduced corneal staining, increased tearing (per the Schirmer I test), reduced inflammatory cytokine levels, increased goblet cell densities, and reduced HLA-DR expression have been documented [3, 4]. Cyclosporine 0.05% was reported to be an effective and safe treatment for patients with primary Sjögren's syndrome (SS)-associated DED [5, 6]. In one study, a cyclosporine 0.05% nanoemulsion improved ocular surface staining scores faster than the conventional cyclosporine 0.05% emulsion [7]. Pretreatment with preservative-free fluorometholone 0.1% eye drops and subsequent switching to cyclosporine 0.05% eye drops was effective in decreasing ocular inflammation and increasing antioxidant contents in patients with dry eye syndrome [8, 9]. In patients with moderate to severe DED who were unresponsive to cyclosporine 0.05%, shifting to cyclosporine 0.1% improved objective signs, albeit with lower treatment tolerability, in the short term [10]. In patients with SS-associated DED, switching from cyclosporine 0.05% to cyclosporine 0.1% improved ocular symptoms and conjunctival staining [11].

However, to the best of our knowledge, no report on the effects of cyclosporine 0.1% after various pretreatments in patients with DED has been published.

Thus, the objective of this study was to compare the clinical outcomes and the effect of cyclosporine 0.1% after no, fluorometholone 0.1%, or cyclosporine 0.05% pretreatment in patients with DED, specifically SS-associated DED.

## 2. Methods

We performed a retrospective chart review and data analysis in compliance with institutional review board regulations and the Declaration of Helsinki. The Institutional Review Board (IRB)/Ethics Committee of Bucheon St. Mary's Hospital approved this study protocol (HC23RASI0113).

### 2.1. Inclusion and Exclusion Criteria

Two hundred seventy-four eyes of 137 patients diagnosed with DED were enrolled at Bucheon St. Mary's Hospital. Patients with the following conditions were excluded: those taking medicine for a systemic disease such as diabetes mellitus, hypertension, allergic disease, or lid abnormalities; those who had undergone ocular surgery within 6 months of the study; those actively using contact lenses or punctual plugs; and those who used topical cyclosporine.

### 2.2. Patient Examination

The enrolled patients were divided into three groups: Group 1 (30 patients with 60 eyes not pretreated), Group 2 (68 patients with 136 eyes pretreated with fluorometholone 0.1%) (Humeron; Hanlim Inc., Seoul, Korea), and Group 3 (39 patients with 78 eyes pretreated with cyclosporine 0.05%) (Restasis; Allergan Inc., Irvine, CA, USA). In Groups 2 and 3, treatment was later switched to cyclosporine 0.1% (Ikerbis; Santen Inc., Osaka, Japan). All patients' demographic and examination data were recorded after a complete ophthalmological examination. The Ocular Surface Disease Index Questionnaire (OSDI) score, Schirmer I test result, noninvasive tear film break-up time (NItBUT), corneal and conjunctival staining score, and matrix metalloproteinase-9 (MMP-9) expression were examined before treatment and at 1, 2, and 3 months after treatment. Corneal and conjunctival staining scores were calculated on the Oxford scale (0–5 points) [12]. The InflammaDry test was used to grade the expression of MMP-9 [13].

### 2.3. Meibomian Gland Examination

Meibomian gland dropout was examined using the Keratograph 5M system (Oculus GmbH, Wetzlar, Germany) and was graded from 0 to 3 points as previously reported [14]. The meibum expression score and meibum quality score were also calculated from 0 to 3 points as previously reported [15].

**Figure 1:**
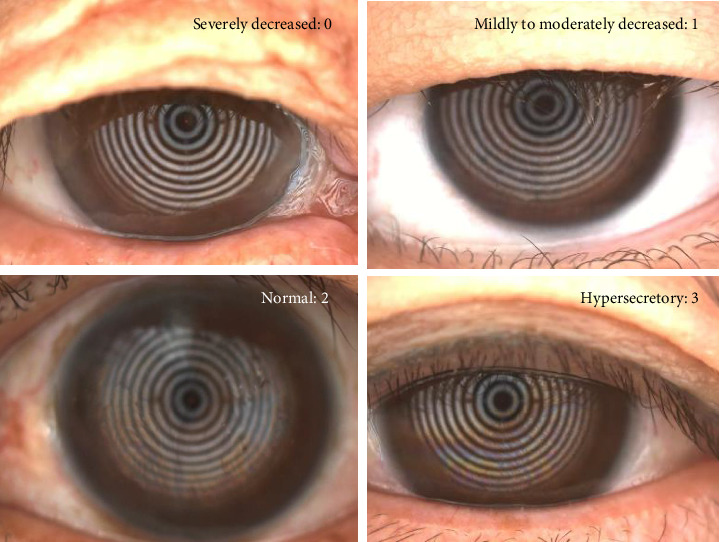
Lipid layer thickness was captured using keratograph 5M. Lipid layer thickness was graded from 0 to 3, as follows: 0, severely decreased lipid layer; 1, mildly to moderately decreased lipid layer; 2, normal lipid layer; and 3, hypersecretary lipid layer.

Lipid layer thickness was captured by the Keratograph 5M system and graded from 0 to 3 points, as follows: 0 points, severely decreased lipid layer; one point, mildly to moderately decreased lipid layer; two points, normal lipid layer; and three points, hypersecretory lipid layer (Figure 1).

### 2.4. Statistical Analysis

All statistical analyses were performed using a commercial program (SPSS for Windows, version 21.0.1; IBM Corporation, Armonk, NY, USA). We calculated a sample size of 137 patients before the study to assess signs and symptoms in patients with DED. The Wilcoxon signed-rank test was used to compare data collected before and after treatment. Comparisons among the three groups were performed using one-way analysis of variance with a Bonferroni post hoc comparison. If the variances were not homogeneous (checked by the test of Levene), the Tamhane test was used for the post hoc comparison between pairs of groups. The chi-square test was used for the comparison between groups of different proportions. *p* < 0.05 was considered statistically significant.

## 3. Results

The expression of MMP-9, the Schirmer I test result, and the corneal and conjunctival fluorescein staining scores in Group 3 were significantly worse than those in Groups 1 and 2 (*p* < 0.05). There were no significant differences between the two groups in age, OSDI score, tear meniscus height, meibomian gland score, or lipid layer thickness (*p* > 0.05). There were 3 (10%), 10 (14.7%), and 10 (25.6%) SS cases in Groups 1, 2, and 3, respectively (Table 1).

### 3.1. OSDI Scoring

**Figure 2:**
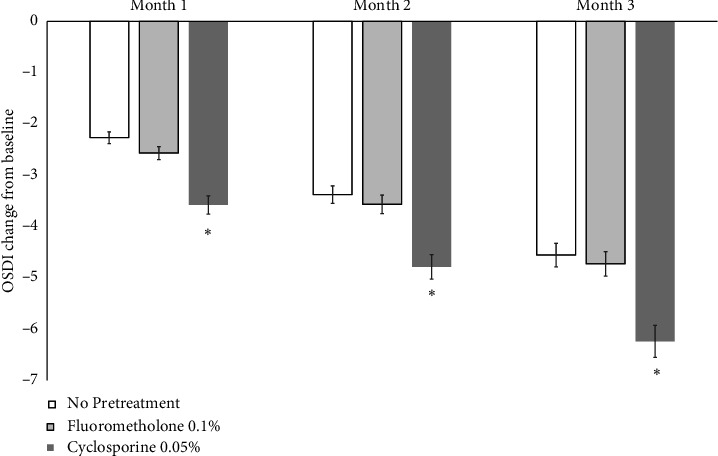
OSDI score change of both groups after treatment. Ocular Surface Disease Index Questionnaire (OSDI) score change from baseline in Group 3 was significantly decreased compared with that in Groups 1 and 2 at 1, 2, and 3 months after the treatment of cyclosporine 0.1%, respectively (*p* < 0.05).

The OSDI scores of all Groups at 1, 2, and 3 months after treatment with cyclosporine 0.1% were significantly decreased compared to those before treatment (*p* < 0.05). The OSDI score from baseline in Group 3 (−3.58 ± 0.45, −3.79 ± 0.84, and −6.45 ± 1.17 points, respectively) was significantly decreased compared to those in Group 1 (−2.27 ± 0.74, −3.38 ± 0.87, and −4.56 ± 1.07 points) and Group 2 (−2.34 ± 0.85, −3.57 ± 0.95, and −4.73 ± 1.12 points) at 1, 2, and 3 months after treatment with cyclosporine 0.1% (*p* < 0.05) (Figure 2).

### 3.2. Schirmer I Test Result and NItBUT

**Figure 3:**
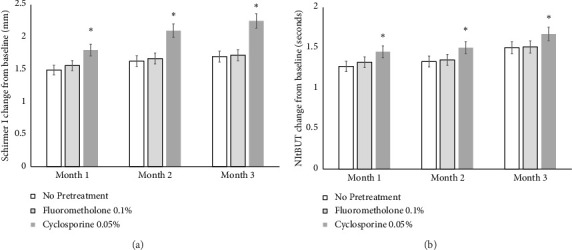
Schirmer I and NItBUT score changes of both groups after treatment. Schirmer I (a) and noninvasive tear break up time (NItBUT) (b) change from baseline in Group 1 were significantly increased compared with those in Groups 1 and 2 at 1, 2, and 3 months after the treatment of cyclosporine 0.1%, respectively (*p* < 0.05).

Both the Schirmer I test result and NItBUT of all Groups at 1, 2, and 3 months after treatment with cyclosporine 0.1% were significantly improved relative to the values recorded before treatment (*p* < 0.05). The Schirmer I test result change from baseline in Group 3 (1.80 ± 0.76, 2.10 ± 0.95, and 2.25 ± 1.08 mm, respectively) was significantly increased compared to those in Group 1 (1.49 ± 0.68, 1.63 ± 0.81, and 1.70 ± 0.89 mm) and Group 2 (1.56 ± 0.85, 1.67 ± 0.91, and 1.72 ± 1.10 mm) at 1, 2, and 3 months after treatment with cyclosporine 0.1% (*p* < 0.05) (Figure 3(a)). The NItBUT change from baseline in Group 3 (1.45 ± 0.60, 1.50 ± 0.79, and 1.67 ± 0.84 s, respectively) was significantly increased compared to those in Group 1 (1.27 ± 0.50, 1.33 ± 0.63, and 1.50 ± 0.78 s) and Group 2 (1.32 ± 0.45, 1.35 ± 0.57, and 1.51 ± 0.83 s) at 1, 2, and 3 months after treatment with cyclosporine 0.1% (*p* < 0.05) (Figure 3(b)).

### 3.3. Corneal and Conjunctival Staining and MMP-9

**Figure 4:**
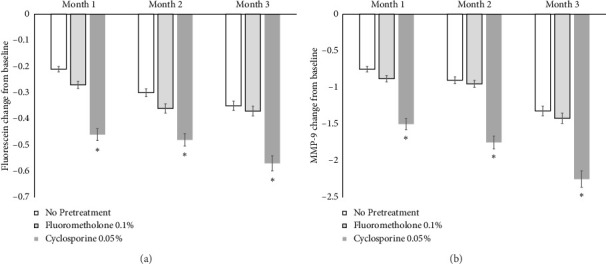
Fluorescein score and MMP-9 grade change of both groups after treatment. Corneal and conjunctival fluorescein score (a) and MMP-9 grade (b) change from baseline in Group 3 were significantly decreased compared with those in Groups 1 and 2 at 1, 2, and 3 months after the treatment of cyclosporine 0.1%, respectively (*p* < 0.05).

The corneal and conjunctival fluorescein score and MMP-9 grade of all groups at 1, 2, and 3 months after treatment were significantly decreased compared to those recorded before treatment with cyclosporine 0.1% (*p* < 0.05). The corneal and conjunctival fluorescein score change from baseline in Group 3 (−0.46 ± 0.12, −0.48 ± 0.15, and −0.57 ± 0.21 points, respectively) was significantly decreased compared to those of Group 1 (−0.21 ± 0.08, −0.30 ± 0.15, and −0.35 ± 0.18 points) and Group 2 (−0.27 ± 0.14, −0.36 ± 0.17, and −0.37 ± 0.20 points) at 1, 2, and 3 months after treatment with cyclosporine 0.1% (*p* < 0.05) (Figure 4(a)). The MMP-9 grade change from baseline in Group 3 (−1.50 ± 0.58, −1.75 ± 0.61, and −2.25 ± 0.82 s, respectively) was also significantly decreased compared to those in Group 1 (−0.75 ± 0.33, −0.90 ± 0.42, and −1.32 ± 0.51 s) and Group 2 (−0.88 ± 0.36, −0.95 ± 0.35, and −1.42 ± 0.57 s) at 1, 2, and 3 months after treatment with cyclosporine 0.1% (*p* < 0.05) (Figure 4(b)).

### 3.4. Meibomian Gland Score

**Figure 5:**
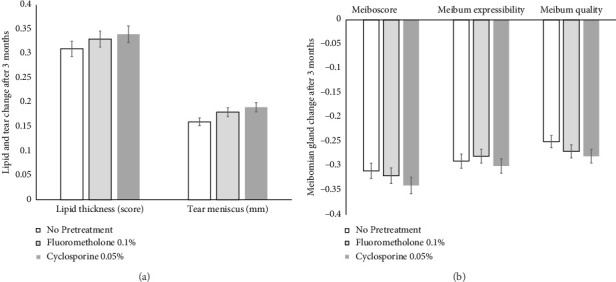
Meibomian gland, lipid thickness, and tear height change after treatment. There was no significant difference in meiboscore, meibum expressibility, meibum quality (a), lipid thickness grade, or tear meniscus height (b) between both groups at 3 months after treatment (*p* > 0.05).

The meiboscore, meibum expressibility, and meibum quality of all groups at 3 months after treatment with cyclosporine 0.1% were significantly improved compared to those recorded before cyclosporine 0.1% treatment (*p* < 0.05). However, there was no significant difference in meiboscore, meibum expressibility, or meibum quality among all groups at 3 months after treatment with cyclosporine 0.1% (*p* > 0.05) (Figure 5(a)).

### 3.5. Lipid Thickness and Tear Meniscus Height

Both the lipid thickness grade and tear meniscus height (mm) of all groups at 3 months after treatment with cyclosporine 0.1% were significantly improved compared to those recorded before treatment (*p* < 0.05). Conversely, there was no significant difference in lipid thickness grade or tear meniscus height among all groups at 3 months after treatment with cyclosporine 0.1% (*p* > 0.05) (Figure 5(b)).

### 3.6. Treatment Discontinuation

The percentages of patients who discontinued cyclosporine 0.1% treatment in Groups 1, 2, and 3 were 20.0% (6 patients), 7.4% (5 patients), and 10.3% (4 patients), respectively. Reasons for discontinuing cyclosporine 0.1% treatment in all groups were ocular pain (*n* = 10), conjunctival hyperemia (*n* = 2), and tearing (*n* = 3) (Table 2).

## 4. Discussion

Dry eye has been defined as a multifactorial disorder that can be classified as either aqueous-deficient or excessive evaporation [6]. Recent observations show that both inflammation and apoptosis have crucial roles in dry eye pathogenesis [1]. Treatment of DED is complicated due to the vicious cycle of tear film instability, hyperosmolarity, and ocular surface inflammation that drives the condition [16, 17]. Because inflammation plays a particularly critical role in the pathogenesis of DED, numerous anti-inflammatory eye drops have been produced [18]. Cyclosporine A (CsA), a calcineurin inhibitor, is used to treat ocular surface inflammatory disorders by inhibiting T-cell activity and inflammatory cytokine expression [3, 19, 20]. CsA also improves signs and symptoms in moderate to severe DED [21].

In the literature, topical CsA was reported to increase corneal sub-basal nerve density, improving clinical signs and symptoms of dry eye associated with SS [22]. Supra-lacrimal protein-based carriers for CsA were reported to decrease Th17-mediated autoimmunity in a murine model of SS [23].

In one study, an oil-based CsA formulation (anionic emulsion (AE)) had low bioavailability and was not well tolerated [24]. Topical CsA 0.05% produced in nanoemulsion form increases the drug bioavailability with poor aqueous solubility [7]. Both nanoemulsion and oil-based CsA improve ocular signs, symptoms, and conjunctival inflammation; however, the CsA nanoemulsion improved ocular surface staining scores faster than the oil-based emulsion [7]. Elsewhere, a CsA 0.1% cationic emulsion (CE) increased the retention time of CsA on the cornea and conjunctiva by interacting with cationic surfactants and the negatively charged mucin in the tear film [25]. A CsA CE also significantly improved signs and symptoms in patients with moderate to severe DED, including patients with SS [21]. However, in patients with moderate to severe DED unresponsive to cyclosporine 0.05%, shifting to cyclosporine 0.1% facilitated improvements in objective signs but with lower treatment tolerability in the short term [10]. In our study, the percentages of patients discontinuing treatment with cyclosporine 0.1% in the no pretreatment, fluorometholone 0.1% pretreatment, and cyclosporine 0.05% pretreatment groups, respectively, were 20.0% (6 patients), 7.4% (5 patients), and 10.3% (4 patients) (Table 2). Treatment with preservative-free fluorometholone 0.1% eye drops in the first month before switching to cyclosporine 0.05% in the second and third months was also reported to decrease ocular inflammation and increase antioxidant contents in the tears of patients with dry eye syndrome [8]. Switching from fluorometholone 0.1% or cyclosporine 0.05% as pretreatment can also increase patient compliance with using cyclosporine 0.1%.

In patients with SS-associated DE, switching from a CsA AE to CsA CE improved ocular symptoms and conjunctival staining. Corneal staining also improved with this approach in patients with severe keratitis [11]. MMP-9 is an inflammatory molecule that participates in the physiological and pathologic mechanisms of dry eye. MMP-9 accelerates corneal epithelial healing by modulating the inflammatory response [26]. In one study of DED patients, MMP-9 expression and grade significantly decreased after treatment with cyclosporine compared to after treatment with diquafosol [13].

In the present study, all dry eye signs and symptoms of all patients at 1, 2, and 3 months were significantly improved compared to those recorded before treatment with cyclosporine 0.1% (*p* < 0.05). The OSDI score, Schirmer I test result, NItBUT, corneal and conjunctival fluorescein score, and MMP-9 grade of Group 3 were significantly improved compared to those of Groups 1 and 2 at 1, 2, and 3 months after treatment with cyclosporine 0.1% (*p* < 0.05) (Figures 2, 3, and 4). However, there was no significant difference in the meiboscore, meibum expressibility, meibum quality, lipid thickness, or tear meniscus height among all groups at 3 months after treatment (*p* > 0.05) (Figure 5).

In this study, the numbers of SS cases in Groups 1, 2, and 3 were 3 (10%), 10 (14.7%), and 10 (25.6%), respectively (Table 1). All dry eye signs and symptoms of SS patients in Group 3 were significantly improved compared to those of SS patients in Groups 1 and 2 because treatment with cyclosporine 0.1% after cyclosporine 0.05% pretreatment improved the signs and symptoms of DED among SS patients. In this study, pretreatment with cyclosporine 0.05% was more effective than pretreatment with fluorometholone 0.1% because the effect of cyclosporine was more durable, with fewer recurrences of an anti-inflammatory response [27]. Because prolonged use of corticosteroids has been associated with intraocular pressure elevation and cataract formation, cyclosporine is preferred over the long term and fluorometholone is preferred in the short term [27].

We initially administered cyclosporine 0.05% to all groups because Group 3 patients were diagnosed with moderate to severe DED. We also switched from cyclosporine 0.05% to cyclosporine 0.1% in Group 3 because Group 3 patients were refractory to topical cyclosporine 0.05%. In other words, Group 3 patients had more severe initial signs and symptoms of dry eye compared to patients in Groups 1 and 2 (Table 1). To decease bias according to the poor baseline scores in Group 3, we adjusted the Group 3 data. Thus, we explain the changes in some scores and factors to be statistically significant because of the poor baseline scores in Group 3. Because of the retrospective nature of this study, our three groups had different initial characteristics. Of note, the number of SS cases in Groups 1, 2, and 3 varied (3 [10%], 10 [14.7%], and 10 [25.6%], respectively). Therefore, we hypothesized that sequential treatments during the shift from cyclosporine 0.05% to cyclosporine 0.1% can improve inflammatory symptoms and signs more so than adopting other treatment strategies in patients with moderate to severe dry eye.

Several studies have reported the efficacy of CsA 0.1% in patients with DED. However, to the best of our knowledge, no study has examined the effect of cyclosporine 0.1% after various pretreatments in patients with DED.

In conclusion, cyclosporine 0.05% pretreatment can improve the effect of cyclosporine 0.1% by better decreasing ocular inflammatory signs and symptoms compared to fluorometholone 0.1% and no pretreatment. Adopting a steroid or lower concentration of cyclosporine eye drops as pretreatment can also increase the compliance of patients with using cyclosporine 0.1% eye drops compared to no pretreatment.

### 4.1. Study Limitations

The limitations of our investigation were that it was a single-center clinical trial with a small sample size and a short follow-up period. Additional research is necessary to observe the long-term efficacy of cyclosporine 0.1% after various pretreatments in patients with DED.

## Figures and Tables

**Table 1:** Data of patients before cyclosporine 0.1% treatment.

Parameter	No pretreatment (Group 1)	Fluorometholone 0.1% (Group 2)	Cyclosporine 0.05% (Group 3)
Number of patients	30	68	39
F:M	23:7	24:6	25:5
Age (years)	57.74 ± 12.03	55.93 ± 12.49	50.01 ± 10.72
OSDI score	36.78 ± 10.12	39.25 ± 11.25	42.38 ± 12.43
Tear height (mm)	0.26 ± 0.10	0.27 ± 0.14	0.22 ± 0.10
MMP-9 grade	0.71 ± 1.13	0.70 ± 0.99	^∗^1.29 ± 0.52
Schirmer I (mm)	4.70 ± 2.17	4.57 ± 2.11	^∗^3.17 ± 1.99
NItBUT (sec)	6.90 ± 4.02	6.68 ± 4.20	6.37 ± 4.17
Fluorescein stain	2.19 ± 1.67	2.18 ± 1.71	^∗^3.35 ± 2.13
Meiboscore	1.13 ± 0.81	1.06 ± 0.84	1.17 ± 0.96
Meibum expressibility	1.19 ± 0.74	1.42 ± 0.81	1.46 ± 0.79
Meibum quality	1.23 ± 0.48	1.36 ± 0.52	1.40 ± 0.64
Lipid layer thickness	0.88 ± 0.96	0.98 ± 0.93	1.26 ± 1.06
No of Sjögren's syn. (%)	3 (10%)	10 (14.7%)	10 (25.6%)

*Note:* The expression of matrix metalloproteinase-9 (MMP-9), Schirmer I test, and corneal and conjunctival fluorescein staining scores in Group 3 were significantly worse than those in Groups 1 and 2, respectively (*p* < 0.05). Fluorescein stain: corneal and conjunctival fluorescein staining score. Values are presented as mean ± SD. D; diopter.

Abbreviations: MMP-9, matrix metalloproteinase-9; NItBUT, noninvasive tear break up time; OSDI, Ocular Surface Disease Index Questionnaire.

^∗^: < 0.05.

**Table 2:** Percentage of discontinued treatment.

Parameter	No pretreatment (Group 1)	Fluorometholone 0.1% (Group 2)	Cyclosporine 0.05% (Group 3)
Number of patients	30	68	39
Discontinued treatment (*N*)	6	5	4
Percentage of discontinued treatment	20.0%	7.4%	10.3%

*Note:* The percentage of patients discontinuing the treatment of cyclosporine 0.1% in Groups 1, 2, and 3 was 20.0% (6 patients), 7.4% (5 patients), and 10.3% (4 patients), respectively.

## Data Availability

The data used to support the findings of this study are available from the corresponding author upon request.

## References

[1] Craig J. P., Nichols K. K., Akpek E. K. (2017). TFOS DEWS II Definition and Classification Report. *Ocular Surface*.

[2] Hyon J. Y., Kim H. M., Lee D. (2014). Korean Guidelines for the Diagnosis and Management of Dry Eye: Development and Validation of Clinical Efficacy. *Korean Journal of Ophthalmology*.

[3] Sall K., Stevenson O. D., Mundorf T. K., Reis B. L. (2000). Two Multicenter, Randomized Studies of the Efficacy and Safety of Cyclosporine Ophthalmic Emulsion in Moderate to Severe Dry Eye Disease. CsA Phase 3 Study Group. *Ophthalmology*.

[4] Kunert K. S., Tisdale A. S., Stern M. E., Smith J. A., Gipson I. K. (2000). Analysis of Topical Cyclosporine Treatment of Patients With Dry Eye Syndrome: Effect on Conjunctival Lymphocytes. *Archives of Ophthalmology*.

[5] Gao M., Zhao L., Liang R., Zhu Q., Zhao Q., Kong X. (2023). Evaluation of the Efficacy and Safety of Topical 0.05% Cyclosporine Eye Drops (II) in the Treatment of Dry Eye Associated With Primary Sjögren’s Syndrome. *Ocular Immunology and Inflammation*.

[6] (2007). The Definition and Classification of Dry Eye Disease: Report of the Definition and Classification Subcommittee of the International Dry Eye WorkShop (2007). *Ocular Surface*.

[7] Kang M. J., Kim Y. H., Chou M. (2020). Evaluation of the Efficacy and Safety of A Novel 0.05% Cyclosporin A Topical Nanoemulsion in Primary Sjögren’s Syndrome Dry Eye. *Ocular Immunology and Inflammation*.

[8] Jee D., Park S. H., Kim M. S., Kim E. C. (2014). Antioxidant and Inflammatory Cytokine in Tears of Patients With Dry Eye Syndrome Treated With Preservative-Free Versus Preserved Eye Drops. *Investigative Ophthalmology and Visual Science*.

[9] Jee D., Park M., Lee H. J., Kim M. S., Kim E. C. (2015). Comparison of Treatment With Preservative-Free Versus Preserved Sodium Hyaluronate 0.1% and Fluorometholone 0.1% Eyedrops After Cataract Surgery in Patients With Preexisting Dry-Eye Syndrome. *Journal of Cataract and Refractive Surgery*.

[10] Chan Y. H., Sun C. C. (2023). Efficacy and Safety of Topical Cyclosporine 0.1% in Moderate-To-Severe Dry Eye Disease Refractory to Topical Cyclosporine 0.05% Regimen. *Taiwan J Ophthalmol*.

[11] Kim J., Moon T. K., Yoon H. J., Ji Y. S., Yoon K. C. (2021). Efficacy of Switching From Cyclosporine A 0.05% Anionic Emulsion to Cyclosporine A 0.1% Cationic Emulsion in Patients With Dry Eye Associated With Sjögren’s Syndrome. *Journal of Ocular Pharmacology and Therapeutics*.

[12] Bron A. J., Evans V. E., Smith J. A. (2003). Grading of Corneal and Conjunctival Staining in the Context of Other Dry Eye Tests. *Cornea*.

[13] So H. R., Baek J., Lee J. Y., Kim H. S., Kim M. S., Kim E. C. (2023). Comparison of Matrix Metallopeptidase-9 Expression Following Cyclosporine and Diquafosol Treatment in Dry Eye. *Annals of Medicine*.

[14] Srinivasan S., Menzies K., Sorbara L., Jones L. (2012). Infrared Imaging of Meibomian Gland Structure Using a Novel Keratograph. *Optometry and Vision Science*.

[15] Chan H. H. (2011). The Effectof Two-Zone Concentric Bifocal Spectacle Lenses on Refractive Error Development and Eye Growth in Young Chicks. *Graefes Archive for Clinical and Experimental Ophthalmology*.

[16] Baudouin C., Aragona P., Messmer E. M. (2013). Role of Hyperosmolarity in the Pathogenesis and Management of Dry Eye Disease: Proceedings of the OCEAN Group Meeting. *Ocular Surface*.

[17] Baudouin C., Irkeç M., Messmer E. M. (2018). Clinical Impact of Inflammation in Dry Eye Disease: Proceedings of the ODISSEY Group Meeting. *Acta Ophthalmologica*.

[18] Kuklinski E., Asbell P. A. (2017). Sjogren’s Syndrome from the Perspective of Ophthalmology. *Clinical Immunology*.

[19] Brignole F., Pisella P. J., De Saint Jean M., Goldschild M., Goguel A., Baudouin C. (2001). Flow Cytometric Analysis of Inflammatory Markers in KCS: 6-Month Treatment With Topical Cyclosporin A. *Investigative Ophthalmology and Visual Science*.

[20] Foulks G. N. (2006). Topical Cyclosporine for Treatment of Ocular Surface Disease. *International Ophthalmology Clinics*.

[21] Leonardi A., Messmer E. M., Labetoulle M. (2019). Efficacy and Safety of 0.1% Ciclosporin A Cationic Emulsion in Dry Eye Disease: A Pooled Analysis of Two Double-Masked, Randomised, Vehicle-Controlled Phase III Clinical Studies. *British Journal of Ophthalmology*.

[22] Levy O., Labbé A., Borderie V. (2017). Increased Corneal Sub-Basal Nerve Density in Patients With Sjögren Syndrome Treated With Topical Cyclosporine A. *Clinical and Experimental Ophthalmology*.

[23] Guo H., Ju Y., Choi M. Supra-Lacrimal Protein-Based Carriers for Cyclosporine A Reduce Th17-Mediated Autoimmunity in Murine Model of Sjögren’s Syndrome. *Biomaterials*.

[24] Lallemand F., Felt-Baeyens O., Besseghir K., Behar-Cohen F., Gurny R. (2003). Cyclosporine A Delivery to the Eye: A Pharmaceutical Challenge. *European Journal of Pharmaceutics and Biopharmaceutics*.

[25] Boboridis K. G., Konstas A. G. P. (2018). Evaluating the Novel Application of Cyclosporine 0.1% in Ocular Surface Disease. *Expert Opinion on Pharmacotherapy*.

[26] Mohan R., Chintala S. K., Jung J. C. (2002). Matrix Metalloproteinase Gelatinase B (MMP-9) Coordinates and Effects Epithelial Regeneration. *Journal of Biological Chemistry*.

[27] Gouider D., Khallouli A., Maalej A. (2021). Corticosteroids Versus Cyclosporine for Subepithelial Infiltrates Secondary to Epidemic Keratoconjunctivitis: A Prospective Randomized Double-Blind Study. *Cornea*.

