# Correction to “Comparison of the Effect of and Compliance With Cyclosporine 0.1% After Various Pretreatments in Dry Eye Disease”

**DOI:** 10.1155/joph/9794683

**Published:** 2026-09-01

D. Jee, S. Y. Han, H. S. Kim, and E. C. Kim, “Comparison of the Effect of and Compliance With Cyclosporine 0.1% After Various Pretreatments in Dry Eye Disease,” *Journal of Ophthalmology*, 2025, no. 1 (2025): 1–8, https://doi.org/10.1155/joph/6744482.

In the article titled ‘Comparison of the Effect of and Compliance With Cyclosporine 0.1% After Various Pretreatments in Dry Eye Disease’ there were errors in the authors’ affiliations. In the corrected affiliations the addition of ‘the’ is required to accurately reflect the institution of The Catholic University of Korea. Additionally, affiliation 1 and 2 require a correction for the associated city, Seoul.

The corrected affiliations are:

^1^Department of Ophthalmology, St. Vincent’s Hospital, College of Medicine, The Catholic University of Korea, Seoul, Republic of Korea

^2^Department of Ophthalmology, Bucheon St. Mary’s Hospital, College of Medicine, The Catholic University of Korea, Seoul, Republic of Korea

^3^Department of Ophthalmology, Seoul St. Mary’s Hospital, College of Medicine, The Catholic University of Korea, Seoul, Republic of Korea

We apologize for this error.

